# Faculty attitudes about interprofessional education

**DOI:** 10.3402/meo.v21.32065

**Published:** 2016-06-27

**Authors:** Gary L. Beck Dallaghan, Erin Hoffman, Elizabeth Lyden, Catherine Bevil

**Affiliations:** 1 Office of Medical Education, College of Medicine, University of Nebraska, Omaha, NE, USA; 2 Physician Assistant Program, College of Allied Health Professions, University of Nebraska, Omaha, NE, USA; 3 Department of Biostatistics, College of Public Health, University of Nebraska, Omaha, NE, USA; 4 Continuing Nursing Education Office, College of Nursing, University of Nebraska, Omaha, NE, USA

**Keywords:** interprofessional education, faculty, attitudes, barriers

## Abstract

**Background:**

Interprofessional education (IPE) is an important component to training health care professionals. Research is limited in exploring the attitudes that faculty hold regarding IPE and what barriers they perceive to participating in IPE. The purpose of this study was to identify faculty attitudes about IPE and to identify barriers to participating in campus-wide IPE activities.

**Methods:**

A locally used questionnaire called the Nebraska Interprofessional Education Attitudes Scale (NIPEAS) was used to assess attitudes related to interprofessional collaboration. Questions regarding perceived barriers were included at the end of the questionnaire. Descriptive and non-parametric statistics were used to analyze the results in aggregate as well as by college. In addition, open-ended questions were analyzed using an immersion/crystallization framework to identify themes.

**Results:**

The results showed that faculty had positive attitudes of IPE, indicating that is not a barrier to participating in IPE activities. Most common barriers to participation were scheduling conflicts ($\chi_{4,285}^{2}$=19.17, *p*=0.001), lack of department support ($\chi_{4,285}^{2}$=10.09, *p*=0.039), and lack of awareness of events ($\chi_{4,285}^{2}$=26.38, *p*=0.000). Narrative comments corroborated that scheduling conflicts are an issue because of other priorities. Those who commented also added to the list of barriers, including relevance of the activities, location, and prior negative experiences.

**Discussion:**

With faculty attitudes being positive, the exploration of faculty's perceived barriers to IPE was considered even more important. Identifying these barriers will allow us to modify our IPE activities from large, campus-wide events to smaller activities that are longitudinal in nature, embedded within current curriculum and involving more authentic experiences.

Interprofessional education (IPE) has become a pervasive component in the dialog of competent and quality health care education (1–3), and research indicates interprofessional teams improve patient outcomes. For health care providers to become competent and quality-focused, prepared to work in interprofessional teams, they should obtain training embedded within health care education curriculum (1–4). The interprofessional core competencies published in 2011 by the Interprofessional Education Collaborative (IPEC) created objective measures for IPE activities and were among the driving forces for agencies that accredit health professions educational programs to establish standards for IPE (5, 6). While accreditation standards are insufficient to hold programs accountable for the full breadth of necessary IPE, their inclusion sends a call to even the most reluctant universities and educators to answer (6).

The delivery of IPE requires multiple levels of support within educational institutions, from administration to faculty (1, 7, 8). While administration provides the framework, it is the faculty who provides the front line instruction of IPE (1). Educators that embrace the new challenge and lead curriculum development reap benefits of networking opportunities, strengthened relationships, and new insights (9). Despite documented benefits, the logistics and time constraints placed on faculty may negatively affect educator attitudes toward IPE (9). Negative attitudes held by faculty can be communicated to students through unconscious cues and non-verbal behavior, resulting in altered perceptions of IPE in students, potentially undermining the intended objectives of the activity (10). Negative attitudes within the faculty across a campus may also lead to lack of participation which can threaten implementation or the maintenance and advancement of interprofessional educational programs (9, 11).

While research emphasizes the importance of assessing both student and faculty attitudes prior to curricular change (12), the research specific to IPE has focused mainly on the need, the outcomes, and the processes of IPE. There remains a lack of research about faculty attitudes regarding IPE. Previous research is limited to faculty attributes that lead to positive attitudes toward IPE (11). In order to manage the faculty attitudes and perceived barriers, universities, administrators, and educators must understand the attitudes of faculties and identify the barriers that exist.

The leadership at a Midwestern medical school created an IPE Curriculum Committee in response to the growing body of research supporting IPE (1–3, 5). This committee developed two programs for year 1 health sciences students in the colleges of allied health professions, medicine, nursing, pharmacy, and public health. Students are divided into small groups with proportional representation from each college in each group. The first event, held during orientation week, focuses on IPEC domains of communication and teamwork (5). The second event, held in February, focuses on IPEC domain of roles and responsibilities. Each event is scheduled for 3 h on a Wednesday afternoon.

A separate subcommittee, tasked with creating effective evaluation tools for IPE activities, was assembled. In addition to program evaluation, the subcommittee developed the Nebraska Interprofessional Education Attitudes Scale (NIPEAS). Each of the 19 items in the instrument relate to specific IPEC core competencies (5). The NIPEAS was originally created and used to assess the health care professional students’ attitudes of IPE activities (13).

After several years of use in student populations, it was decided to expand the use of this internal attitude assessment tool to faculty. Attitudes influence effective collaboration; therefore, we asked faculty to complete the NIPEAS to determine if attitudes potentially created a barrier to participating in IPE activities on campus. As the university proposes new initiatives to expand and develop more intensive interprofessional activities, logistical barriers to participating were also investigated.

## Methods

### Sample

Full-time faculty across campus were invited to participate in the survey. Because we were interested in knowing what barriers faculty experience participating in IPE activities, we extended the invitation to participate to all faculty on campus and not just those who had participated in the activities.

### Survey instrument

The NIPEAS is a 19-item questionnaire assessing attitudes related to interprofessional collaboration. The items are rated from 1=Strongly Agree to 5=Strongly Disagree. The NIPEAS was initially utilized to assess the attitudes of health care professional students both before and after they had participated in interprofessional activities. It was designed to be used not only with students but also practicing health care professionals. Items on the NIPEAS stem from the IPEC Competencies (5). It was decided to expand the use of the NIPEAS to assess the attitudes of the faculty across the university system. Institutional Review Board approval was obtained for this study.

Full-time faculty were sent a link to the NIPEAS questionnaire, which additionally included questions about perceived barriers to participating in interprofessional educational activities. A list of 12 items was provided for them to check. There was also an option to type in additional responses of perceived obstacles.

### Statistical analysis

Frequency statistics were calculated for faculty responses to each item of the NIPEAS questionnaire. Kruskal–Wallis test was conducted to identify differences among colleges for NIPEAS items. Descriptive statistics and Chi-square analysis were calculated for questions about perceived barriers to participating in IPE activities. Analyses were conducted using IBM SPSS 22.

### Thematic analysis

Faculty were asked to provide narrative comments pertaining to perceived barriers to participating in IPE activities. They were also asked to provide suggestions on how to improve IPE on campus. These comments were analyzed for themes using a crystallization/immersion method. Two of the authors reviewed the comments and came to a consensus on their meaning. Four themes that emerged from the analysis were categorized as priorities, relevance, location, and negative experience. Three themes emerged from an analysis of the recommendations: integration, session content, and timing.

## Results

Two-hundred and eighty five faculty representing all colleges on the UNMC Omaha campus completed the survey (52.8% from the College of Medicine). Table 1 shows the distribution from the colleges.

**Table 1:** Faculty participants

Academic unit	Faculty, *n* (%)
Allied health	31 (9.2)
Medicine	178 (52.8)
Nursing	35 (10.4)
Pharmacy	13 (3.9)
Public health	28 (8.3)

### Survey instrument

Faculty responses to the NIPEAS were primarily positive (‘agree’ and ‘strongly agree’) for all items of the NIPEAS (Table 2). The Kruskal–Wallis test showed that there was a statistically significant difference on three of the items. For Item 3, ‘I understand my own role within the health care team’, $\chi_{4,285}^{2}$=19.176, *p*=0.001 with a mean rank for this item of Public Health=159.93, Medicine=124.19, Allied Health=107.02, Nursing=94.78, and Pharmacy=88.38. Item 6, ‘Appreciation of the expertise of other health care professionals leads to a better work environment’, resulted in $\chi_{4,285}^{2}$=9.746, *p*=0.045 with mean rankings of Public Health=134.33, Medicine=122.83, Pharmacy=108.88, Nursing=101.40, and Allied Health=95.68. Item 19, ‘The health care team's approach should be determined by the team as a whole rather than by the team leader’, had a result of $\chi_{4,285}^{2}$=21.023, *p*=0.000, with mean rankings of Medicine=128.97, Public Health=125.69, Allied Health=89.78, Nursing=88.12, and Pharmacy=77.46.

**Table 2:** Responses to NIPEAS items

	Faculty (*n*=280)						
	
	Strongly agree	Agree	Neutral	Disagree	Strongly disagree	*χ* ^2^	*p*
1. I am able to communicate effectively about patient care with persons from health care professions different from my own.	47.84	39.93	8.99	2.16	1.08	6.98	0.137
2. I am able to use terminology that is unique to other health care professions.	23.02	48.20	21.58	6.83	0.36	8.86	0.065
3. I understand my own role within the health care team.	61.15	30.58	5.04	2.88	0.36	**19.2**	**0.001**
4. I understand the roles of other health care professionals.	41.37	47.48	10.43	0.72	.	2.21	0.697
5. I should learn about the values and expertise required for health care professions other than my own.	42.75	41.30	13.41	2.17	0.36	4.20	0.380
6. Appreciation of the expertise of other health care professionals leads to a better work environment.	65.95	28.32	5.02	0.72	.	**9.75**	**0.045**
7. I can provide a higher standard of care if I consider input from other professionals than if I work independently.	60.51	28.62	9.42	1.45	.	8.81	0.066
8. In order to be an effective team member, I may have to compromise with others who hold different values.	37.91	42.96	15.52	3.25	0.36	1.49	0.828
9. To be competent, a person in my profession must work cooperatively with other health care providers.	61.59	28.99	6.52	2.17	0.72	8.30	0.081
10. Ethical principles that are foundational to health care are the same for all health care professions.	49.46	39.35	7.94	2.53	0.72	2.32	0.677
11. I consider ethical practice and high quality of patient care to be more important than demonstrations of my own knowledge and skills.	58.21	27.50	12.14	1.79	0.36	7.92	0.094
12. I would be receptive to critique of my performance from another person in my own profession.	45.32	45.32	7.55	1.08	0.72	1.14	0.888
13. I can learn about my own profession from health care professionals outside of my own profession.	35.48	43.01	16.85	4.30	0.36	7.17	0.127
14. Effective communication is an essential component of all treatment plans.	74.91	22.58	2.15	0.36	.	4.32	0.365
15. I need to establish good relationships with professionals outside of my own profession in order to practice effectively.	57.61	35.14	5.80	1.45	.	2.38	0.667
16. It is more important to listen to the opinions of other health care team members than to state my own viewpoint.	19.06	40.65	29.86	9.71	0.72	3.75	0.441
17. Forming relationships with members of other professions can improve patient care and advance learning.	61.15	35.97	2.88	.	.	5.13	0.274
18. I would be receptive to a critique of my performance from a person who is in a different profession than my own.	31.65	45.32	16.19	4.68	2.16	2.23	0.693
19. The health care team's approach should be determined by the team as a whole rather than by the team leader.	38.13	36.33	19.42	6.12		**21.0**	**0.000**

Bold values indicates level of significance <0.05.

Table 3 shows the most frequent reasons for not participating in IPE activities. A Chi-square test was conducted to determine if there were significant differences across colleges for each item. Scheduling conflicts was significantly different across colleges ($\chi_{4,285}^{2}$=13.91, *p*=0.008) where 69.6% of Medicine faculty reported this problem of the 47.4% who identified this barrier. Lack of departmental support for these teaching activities was significantly different across colleges ($\chi_{4,285}^{2}$=10.09, *p*=0.039). Medicine faculty constituted 79.1% of the 15.1% of respondents identifying this barrier. Nearly 26% of respondents indicated a lack of awareness of the IPE events, which was also statistically different ($\chi_{4,285}^{2}$=26.38, *p*=0.000), with 86.3% of Medicine faculty indicating this as a barrier.

**Table 3:** Frequency of faculty indicating barriers to participation by academic unit

	Medicine	Nursing	Pharmacy	Public health	Allied health	*χ*^2^	*p*
Sessions too long (can't commit the time)	45	4	1	9	7	6.18	0.186
Schedule (clinic conflicts, etc.)	94	12	5	6	18	**13.91**	**0.008**
Competing priorities (workload does not permit a 3-h time commitment)	94	12	4	14	19	7.52	0.111
No experience with collaborative practice	19	3	0	3	0	5.17	0.270
Limited experience facilitating small groups of students	11	0	0	1	0	5.21	0.266
No academic (e.g., workload) credit for teaching non-credit sessions	51	7	4	8	5	3.09	0.543
No support from the department	34	5	0	4	0	**10.09**	**0.039**
Unaware of these events	63	4	1	5	0	26.38	0.000
Perceive these events as not relevant to my program	16	1	0	5	0	9.29	0.054
Time needed to do training prior to the event	22	5	3	8	4	5.87	0.209
Do not feel the offered experience design is of value	13	2	1	4	3	1.97	0.741
Lack of interest	9	1	0	4	0	7.86	0.097

Bold values indicates level of significance <0.05.

### Obstacles for participation in IPE activities

To have greater understanding of the obstacles for paticipation, participants were asked to comment on the struggles they have participating in IPE activities. Analysis of the responses identified four themes, namely priorities, relevance, location, and negative experience.

#### Priorities

This theme was represented in different ways. For some, patient care responsibilities were noted as preventing participation. ‘I do these whenever I can; sometimes, higher priority events supersede my involvement’ (Medicine faculty)**. In addition, other comments indicated this was not a priority of departmental administration, which prevented justifying participation. ‘Chair does not want time out of clinic for these events’ (Medicine faculty).

#### Relevance

Content of the IPE activities held on campus initially focused more on patient care activities. Respondents commented that the sessions do not seem to match their discipline. With faculty involvement from public health and basic science departments in the College of Medicine, the IPE activity topics initially would have made their participation difficult. ‘Not clear how interprofessional education is directly relevant to my own teaching and department’ (Public Health faculty).

#### Location

Faculty who are not at the Omaha campus cannot come to town to participate. ‘I am from Lincoln campus, therefore, we have limited collaborative practice’ (Nursing faculty).

#### Negative experience

Some faculty volunteered in the past and had negative experiences, with medical students being disrespectful. ‘Have previously done this but due to the attitudes of the med students will not do again. They are very disdainful of other students openly speaking about their superiority. Not putting forth effort’ (Nursing faculty). Other faculty noted the event activities were unrealistic and a waste of time. ‘I felt the methods used, the activities offered and the way in which it was done was essentially worthless and did not reflect what the “real world” of interdisciplinary work teams’ (Medicine faculty).

## Discussion

To better understand what factors influence voluntary participation by faculty in IPE activities, we sought to characterize faculty attitudes about IPE using the NIPEAS. In order for IPE activities to succeed, it is important to know if the faculty have favorable attitudes about the concept of IPE and interprofessional collaboration (10). Faculty strongly agreed to 13 of 19 items on the NIPEAS. When investigating attitudes across colleges, three items were statistically different. Faculty in the College of Public Health rated understanding the various roles of the health care teams and appreciation of all team members’ efforts in health care higher than other faculty. Traditionally having been considered the natural team leaders, Medicine faculty actually rated making group decisions higher than the other colleges. Our campus has several services wherein multidisciplinary teams must work together to ensure positive patient care outcomes which may be an underlying explanation for physician attitudes to be so positive, which relates to reports about house officer training (14). In summary attitudes rated as positive; it does not appear that faculty attitudes create a barrier to participation or engagement into IPE activities.

Since attitudes regarding IPE do not appear to be an issue based on the NIPEAS, understanding other potential barriers to participating in IPE activities is even more important. Scheduling conflicts and lack of departmental support were most common barriers identified by College of Medicine faculty. This was reinforced by the emphasis on this not being a priority from the thematic analysis. This could also explain why College of Medicine faculty most frequently indicated they were unaware of IPE activities. With the emphasis on clinical activities, announcements emailed about participating in these events may easily be overlooked. This further reinforces the work at Laval University and the importance of strong prioritization of IPE activities by senior leaders (8).

Although lack of departmental support was only 9.3%, lack of relevance of the topics in these sessions created a greater barrier for faculty in the College of Public Health. The IPE activities incorporate case discussions and if there is not a deliberate inclusion of how public health fits into the discussion, the faculty may not feel they have much to contribute. It should be noted that although public health faculty did question the relevance of the educational events, they recognized the importance of team efforts due to the nature of their profession.

Identifying these barriers will allow us to modify our current IPE activities to better meet the needs of the faculty. IPE program administrators can capitalize on the positive attitudes faculty have toward IPE to shift the campus from large, campus-wide events that occur outside standard curriculum for all academic units to smaller activities that are longitudinal in nature and involve more authentic experiences. That would also facilitate involvement of public health and graduate college faculty. Superimposing IPE activities within the existing curricula can minimize conflicts with competing priorities, lack of academic credit, and scheduling conflicts. Looking for opportunities to engage the different colleges in ways that augment their curriculum would limit, if not eliminate, the barrier of lack of relevance and has the potential to remove the barrier of location as well.

This study is limited by being from a single institution using a locally developed attitude scale. We have collected validity evidence for the NIPEAS and have submitted the results for publication (13). We also recognize that the IPE activities offered are unique to our campus. However, the barriers to participation in IPE activities experienced by the faculty are common problems faced by many institutions. Coming up with creative solutions to give faculty time to role model interprofessional collaboration through relevant and authentic educational activities is vital to the success of future endeavors.

Thoughtful consideration must be given to barriers to participation to further enhance and promote IPE and thereby enhance interprofessional collaboration. Health care in the future involves functioning in teams of providers. Those who train in interprofessional teams will be well prepared to work in interprofessional teams for the benefit of all patients. In order to facilitate this ideal learning environment, faculty barriers need to be addressed so that they can create opportunities for and participate in IPE activities.

## References

[1] Schmitt MH, Gilbert JHV, Brandt BF, Weinstein RS (2013). The coming of age for interprofessional education and practice. Am J Med.

[2] Hammick M, Freeth D, Koppel I, Reeves S, Barr H (2007). A best evidence systematic review of interprofessional education: BEME Guide No. 9. Med Teach.

[3] Institute of Medicine (US) Committee on Quality of Health Care in America (2001). Crossing the quality chasm: a new health system for the 21st century.

[4] Hasnain M, Koronkowski MJ, Kondratowicz DM, Goliak KL (2012). Training future health providers to care for the underserved: a pilot interprofessional experience. Educ Health.

[5] Interprofessional Education Collaborative Expert Panel (2011). Core competencies for interprofessional collaborative practice: report of an expert panel.

[6] Zorek J, Raehl C (2013). Interprofessional education accreditation standards in the USA: a comparative analysis. J Interprof Care.

[7] Dumont S, Brière N, Morin D, Houle N, Iloko-Fundi M (2010). Implementing an interfaculty series of courses on interprofessional collaboration in prelicensure health science curriculums. Educ Health.

[8] Milot É, Dumont S, Aubin M, Bourdeau G, Mbourou Azizah G, Picard L (2015). Building an interfaculty interprofessional education a curriculum: what can we learn from the Université Laval experience?. Educ Health.

[9] Anderson ES, Thorpe LN (2010). Interprofessional educator ambassadors: an empirical study of motivation and added value. Med Teach.

[10] Chuang AW, Nuthalapaty FS, Casey PM, Kaczmarczyk JM, Cullimore AJ, Dalrymple JL (2010). To the point: reviews in medical education-taking control of the hidden curriculum. Am J Obstet Gynecol.

[11] Curran VR, Sharpe D, Forristall J (2007). Attitudes of health sciences faculty members towards interprofessional teamwork and education. Med Educ.

[12] Brynhildsen J, Dahle LO, Behrbohm Fallsberg M, Rundquist I, Hammar M (2002). Attitudes among students and teachers on vertical integration between clinical medicine and basic science within a problem-based undergraduate medical curriculum. Med Teach.

[13] Beck Dallaghan GL, Lyden E, Meza J, Stoddard H, Bevil C, Collier D (2016). The Nebraska Interprofessional Education Attitudes Scale: a new instrument for assessing the attitudes of health professions students. J Interprof Educ Pract. In press, June.

[14] van Schaik S, Plant J, O'Brien B (2015). Challenges of interprofessional team training: a qualitative analysis of residents’ perceptions. Educ Health.

